# Relevance of activated leukocyte cell adhesion molecule (ALCAM) in tumor tissue and sera of cervical cancer patients

**DOI:** 10.1186/1471-2407-12-140

**Published:** 2012-04-04

**Authors:** Maike Ihnen, Kerstin Kress, Jan Felix Kersten, Ergin Kilic, Matthias Choschzick, Hilke Zander, Volkmar Müller, Sven Mahner, Fritz Jänicke, Linn Woelber, Karin Milde-Langosch

**Affiliations:** 1 Department of Gynecology, University Medical Center Hamburg-Eppendorf, Martinistrasse 52, 20246 Hamburg, Germany; 2 Department of Medical Biometry and Epidemiology, University Medical Center Hamburg-Eppendorf, Martinistrasse 52, 20246 Hamburg, Germany; 3 Institute for Pathology, University Medical Center Hamburg-Eppendorf, Martinistrasse 52, 20246 Hamburg, Germany; 4 Department of General, Visceral and Thoracic Surgery, University Medical Center Hamburg-Eppendorf, Martinistrasse 52, 20246 Hamburg, Germany

## Abstract

**Background:**

An altered expression of the activated leukocyte cell adhesion molecule (ALCAM) is associated with cancer progression in various cancer types. In some cancers ALCAM has a prognostic value or is predictive for the benefit of therapeutic interventions. To date there are no data on the role of ALCAM in cervical cancer available.

**Methods:**

In this study, ALCAM expression was analysed by immunohistochemistry (IHC) in tissue samples of 233 patients with cervical cancer, among them 178 with complete follow-up information. In addition, soluble (s-)ALCAM was measured in sera of a subset of the included patients (n = 55) by enzyme-linked immunosorbent assay (ELISA).

**Results:**

ALCAM overexpression was detected (immunoreactive score (IRS) 2-12) in 58.4% of the cervical cancer samples. The normal ectocervical or endocervical epithelium showed no ALCAM reactivity. In untreated patients, ALCAM overexpression in tumor tissue tended to be associated with shorter cancer-specific survival (CSS) and disease-free survival (DFS). Patients, whose tumor samples showed ALCAM overexpression receiving a cytotoxic therapy like radiotherapy or chemoradiation, however, had a favourable prognosis compared to those patients, whose cancers showed no or minimal ALCAM staining. This effect was particularly apparent in patients receiving chemoradiation where the CSS was significantly longer in patients with ALCAM-positive tumors (p = 0.038; cumulative incidence rates at 96 months 8%, 95% CI 0%-23%, and 26%, CI 3%-43% in ALCAM-positive and ALCAM-negative cases, respectively).

Median preoperative s-ALCAM concentration in sera from tumor patients was 27.6 ng/ml (range 17.5-55.1 ng/ml, mean 28.9 ng/ml), serum levels did not correlate with intratumoral ALCAM expression.

**Conclusions:**

The data of our retrospective study suggest that the prognostic value of ALCAM expression in cervical carcinoma might be therapy-dependent, and that ALCAM might function as a predictive marker for the response to chemoradiation. This should be confirmed in further, prospective studies.

## Background

Cervical cancer is the third most common malignancy in women, accounting for 8.8% of all cancers. Worldwide there were estimated 529,000 new cases in 2008 and 274,000 deaths due to cervical cancer [1]. Locally advanced cervical cancers (stage IB2-IVA) are generally treated by primary chemoradiotherapy http://www.nccn.org, http://www.ago-online.org. In early stage disease (FIGO 0 to IB1) and also in stage IIA cancers, treatment guidelines give several treatment choices to the oncologists and therapeutic decisions are often subject to discussion. Therapeutic options include surgery with or without radiotherapy or concomitant chemoradiotherapy. The applied regimens have various side effects, especially when treatment modalities (surgery and chemoradiotherapy) are combined. However, predictive factors for the benefit of chemo- and or radiotherapy in the treatment of this disease remain scarse [2].

ALCAM is a glycoprotein of the immunoglobulin superfamily of adhesion molecules (IgCAMs). IgCAMs are mostly transmembrane proteins, functioning not only as cell adhesion receptors, but also transducing signals to intracellular signalling pathways [3]. ALCAM expression has been described in subsets of cells being involved in dynamic growth and migration but it has also been detected in cancer stem cells [4]. In various neoplasms like malignant melanoma [5], prostate cancer [6], colorectal carcinoma [7], bladder cancer [8] and breast cancer [9] as well as in oral [10] and esophageal squamous cell cancer [11] a pathologically altered ALCAM expression has been observed and was associated with cancer progression. On the other hand, ALCAM expression in tumor tissue has been reported to be a potential marker for the benefit of therapeutical interventions: Previous studies of our group and others could show that ALCAM expression predicted chemotherapy response in early breast cancer [12] and pancreatic cancer cells [13]. The extracellular domain of ALCAM can be shedded by proteases [14]. Recent studies measured soluble ALCAM levels (s-ALCAM) in blood serum of tumor patients, e.g. breast cancer, suggesting a potential value of s-ALCAM as a biomarker for cancer detection [15]. No data is available for the role of ALCAM or s-ALCAM in cervical cancer so far.

In this hypothesis generating study we analysed ALCAM expression in cervical cancer tissue and its correlation with clinico-pathological tumor characteristics. In addition, we examined the prognostic and predictive impact of ALCAM expression in cervical cancer and s-ALCAM expression in sera of a subset of patients. We could demonstrate that ALCAM expression in cervical cancer could function as a marker for improved outcome in patients treated with radiotherapy or chemoradiotherapy.

## Methods

### Patients

Tumor tissue of 233 patients undergoing surgery for primary cervical cancer in 4 different hospitals between 1993 and 2008 was analyzed. For 178 patients detailed follow-up data from the date of primary surgery to the date of death or last contact (July 2009) were available. Patient characteristics are listed in Tables 1 and 2. In addition, 55 preoperative serum samples from a subset of the included patients treated in one participating center were analysed to detect s-ALCAM levels. To assure the analysis of a representative cohort, FIGO stages in this subcohort were compared to those of the whole cohort showing a similar stage distribution (FIGO stage I, 67%; stage II, 24%; stage III/IV, 9%; compare Table 1). Clinicopathologic factors were evaluated by reviewing medical charts and pathologic reports of the department of gynecological pathology at University Medical Center Hamburg-Eppendorf which acted as reference pathology. Informed consent for the scientific use of patient data, tumor tissue and serum had been obtained from all patients in coordination with the local ethics committee (Ethics committee of the Medical Board Hamburg, reference number #190504). All data were analyzed anonymously. The study was performed in accordance to the principles of the declaration of Helsinki and REMARK criteria [16].

**Table 1:** Cohort characteristics

	n	%
***Total cohort (n = 233)***		

***Age***		
**Mean/median age**	23-85 y	
**Range**	49/47 y	

***Tumor stage (FIGO***		
**Ia**	4	(1.7)
**Ib**	139	(59.7)
**IIa**	17	(7.3)
**IIb**	48	(20.6)
**IIIb**	1	(0.4)
**IVa**	7	(3.0)
**IVb**	17	(7.4)

***Postoperative residual tumor***		
**R0**	212	(91.0)
**R1**	21	(9.0)

***Subcohort with follow-up informations (n = 178)***		

***Applied therapy regimen***		
**Observation**	86	(48.3)
**Adjuvant radiotherapy alone**	42	(23.6)
**Adjuvant chemoradiotherapy**	50	(28.0)

***Clinical follow-up***		
**Median follow-up time: 49 months**		
**Recurrence**	47	(25.4)
**Dead of disease (DOD)**	41	(22.2)

**Table 2:** Correlations between ALCAM expression and clinicopathological factors

			ALCAM expression		
	
		All patients	negative (IRS 0-1)	positive (IRS 2-12)	p-value
**All samples**		233	97	136	

*Histological subtype *(n = 233)	squamous	171	70	101	
		
	adenomatous	35	18	17	
		
	adenosquamous	27	9	18	0.338

*FIGO stage *(n = 233)	I	143	60	83	
		
	II	65	25	40	
		
	III/IV	25	12	13	0.971

*Nodal* *involvement *(n = 233)	pN0	175	75	100	
	pN1	58	22	36	0.542

*Number of Positive lymph nodes *(n = 233)	0	175	75	100	
		
	1-3	39	12	27	
		
	> 3	19	10	9	0.229

*Tumor grade* (n = 227)	G1	9	4	5	
		
	G2	100	43	57	
		
	G3	118	46	72	0.788

*Lymphatic invasion *(n = 207)	L0	68	33	35	
		
	L1	139	55	84	0.234

*Invasion depth in mm *(n = 195)	geometric mean	14.2	14.3 (n = 90)	14.1 (n = 105)	0.856

*Patients age in years *(n = 233)	mean	48.8	48.3	49.1	0.662

*SCC-Ag in μg/L *(n = 156)	geometric mean	1.9	2.5 (n = 64)	1.5 (n = 92)	**0.012**

*s-ALCAM in ng/mL *(n = 55)	geometric mean	28.3	29.0 (n = 21)	27.8 (n = 34)	0.515

*Progression-free survival in months (PFS; n = 47)*	median	13	9 (n = 19)	15 (n = 28)	0.208

The treatment of cervical cancer patients during the investigational period consisted of radical hysterectomy and resection of the pelvic and paraaortic lymph nodes using laparotomy for FIGO stages I and II. Only in stage Ia and Ib disease with fertility preserving objective radical hysterectomy was omitted. In cases of advanced disease radio- and/or chemotherapy of the pelvis and the paraaortic region was performed in accordance to German Cancer Guidelines or in clinical trials. Depending on tumor size and nodal involvement mean radiation dose was 50.4 Gy (range 45-55.8 Gy) due to the dose prescription habits of the individual hospital and dose volume histograms.

In our study, 25 patients were included who received primary surgery, even though they had been diagnosed with FIGO stage III/IV disease. Although primary chemoradiation is generally recommended in these cases, surgery had been performed either to prevent further tumor invasion into the bladder and/or rectum or because there were no signs of distant disease apparent before surgery.

In order to analyse the predictive value of ALCAM expression, patients with complete follow-up data (n = 178) were stratified into untreated patients (n = 86), patients who received adjuvant radiotherapy only (n = 42) and patients, who were treated by using a combination of chemotherapy and radiotherapy (chemoradiation; n = 50). Applied chemotherapy in the subgroup with follow-up information included cisplatin as single agent or in combination with (n = 47), carboplatin/ifosfamide (n = 2) or other regimens (n = 1).

### Immunohistochemistry

Tissue processing was identical for all participating laboratories. Fixation was performed by using 4% buffered formalin. For evaluation all samples were sent as paraffin-embedded tissue blocks to the department of gynecological pathology, University Medical Center Hamburg-Eppendorf.

Tissue sections were cut at 5 μm, mounted on slides, dewaxed with xylene and gradually hydrated. Subsequently, the slides were placed in boiling 10 mM citrate buffer for 30 min, washed in Aqua dest., immersed in 0,5% H_2_O_2_-methanol for 30 min and washed again in TRIS buffered saline (TBS). For immunohistochemistry, the Vectastain Elite ABC Kit - Peroxidase (Mouse IgG; Vector Laboratories, Inc., Burlingham, CA, U.S.A.) and the DAB Peroxidase Substrate Kit (Vector Lab.) were applied. Incubation with the primary ALCAM antibody (1:400; Vector Lab.) was performed at 4°C overnight. The slides were briefly counterstained with haematoxylin and dehydrated before mounting. As positive control, an ALCAM-positive mammary tumor according to our own prior IHC and Western blot results was included in each experiment. For negative controls, the primary antibody was omitted (not shown). The staining results were evaluated independently by a gynaecologist and a gynaecological pathologist using the immunoreactive score (IRS) as product of staining intensity (graded between 0 and 3) and percentage of positive cells (0:none, 1: 1-20%, 2: 21-50%; 3: 51-80%, 4: > 80%) resulting in a score from 0-12 [17]. For statistical evaluation the cohort was separated into two groups: One included absent or only focal and punctual ALCAM staining (IRS 0-1; referred "ALCAM-negative"), the second group showed weak to strong ALCAM expression and was assessed as "ALCAM-positive" (IRS 2-12). No separate evaluation of membraneous and cytoplasmic immunostaining was performed.

### Detection of soluble human ALCAM (s-ALCAM) in serum samples

For the detection of s-ALCAM in 55 serum samples of tumor patients, 96-well microtiter plates (Costar 9019) were coated with 50 μl per well of 2 μg/ml of capturing antibody MAB6561 (R&D systems, Minneapolis, Minnesota) overnight at 4°C, and the ELISA reaction was performed as described [18]. Human ALCAM-Fc protein (R&D systems, Minneapolis, Minnesota) served as an internal standard control.

### Statistical analysis

We report frequencies and median/mean values for categorical and continuous variables, respectively. If necessary, continuous variables were log-transformed. The Chi-square test was used to analyze the relation between the ALCAM expression (positive/negative) and categorial variables (lymph node involvement, histologic subtype, invasion into lymphatic or blood vessels, grading and FIGO-stage). Two sample *t*-test was applied to compare continuous variables (age, SCC values and s-ALCAM levels) with regard to ALCAM expression groups and to analyse s-ALCAM levels within different FIGO-stage groups, histologic subtype groups, groups of nodal involvement and occurrence of preoperative distant metastases.

Pearson correlation was applied to compare serum SCC and s-ALCAM levels. The impact of ALCAM expression on OAS (overall survival; time from diagnosis to death of any cause), CSS (cancer-specific survival; time from diagnosis to death due to the cervical tumor) and DFS (disease-free survival; time from diagnosis to relapse) was investigated using Kaplan-Meier analysis and log-rank tests, partly stratified for the applied therapy-regimen. In addition, univariate and multivariate Cox regression analysis was performed. Since 9% of patients in this cohort died of other reasons than cervical carcinoma, we decided to focus on CSS and DFS in statistical calculations. P values < 0.05, two tailed, were considered significant. Statistical analysis was performed using SPSS 15.0 and the Statistical Package R version 2.9.2.

## Results

### ALCAM distribution in cervical cancer and normal cervical tissue

**Figure 1:**
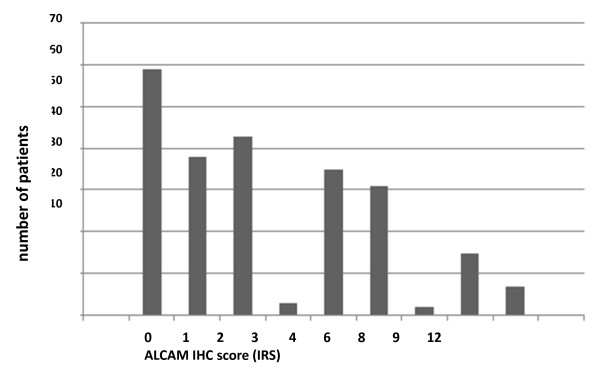
**Distribution of ALCAM immunoreactive scores (IRS) in cervical carcinomas (n. For evaluation of staining intensities: see methods**.

**Figure 2:**
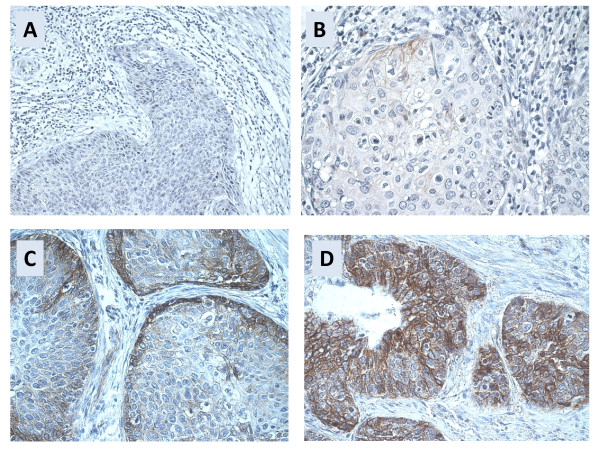
**ALCAM staining patterns in squamous cervical carcinomas**.

Evaluation of ALCAM immunostaining resulted in immunoreactive scores (IRS) from 0 to 12 (Figure 1). ALCAM expression patterns varied from focal staining in specific tumor areas (Figure 2A-B) to ALCAM reactivity in all tumor cells, with both membraneous and cytoplasmic staining in varying proportions (Figure 2D and Additional file 1). Staining intensity also varied from very weak to strong. Interestingly, in most cervical carcinomas which exhibit moderate ALCAM staining levels, ALCAM reactivity seemed to be more accentuated at the invasion front (Figure 2C). The normal surface epithelium of the ectocervix (squamous differentiation) and the endocervix (glandular differentiation) showed no ALCAM reactivity.

A, ALCAM-negative tumor (IRS0; 200×); B, weak and focal membraneous ALCAM staining (IRS1; 400×); C, moderate staining pattern with prominent staining intensity in the periphery of tumor (IRS6; 400×); D, strong ALCAM expression in most tumor cells (IRS9; 200×)

For statistical analysis, we defined two groups according to ALCAM reactivity: An ALCAM-negative group (IRS 0-1), which contains ALCAM-negative tumors and those exhibiting a punctual ALCAM staining and an ALCAM-positive group (IRS 2-12). By using this classification ALCAM staining was positive in 58.4% and negative in 41.6% of all cases.

### Correlations of ALCAM staining with clinical and histological tumor characteristics

ALCAM-positive and ALCAM-negative cervical carcinomas were first compared regarding their clinicopathological markers (Table 2). No correlations between ALCAM expression and the investigated clinicopathological variables could be demonstrated except serum SCC levels which showed a significant inverse correlation with ALCAM expression levels (p = 0.012).

In the subcohort with follow-up information (n = 178), advanced FIGO stage, nodal involvement, invasion depth and lymphatic invasion were associated with significantly increased risk of recurrence or death in univariate analysis (Table 3). In contrast, ALCAM positivity or negativity did not show any association with patients' survival in univariate analysis (Table 3) and Kaplan-Meier analysis (CSS: p = 0.871; DFS: p = 0.755; not shown). In multivariate analysis including stage, grading, nodal status, histological type and ALCAM expression, only advanced stage and nodal involvement turned out as significant indicators of shorter DFS and CSS (Additional file 2).

**Table 3:** Univariate analysis of clinicopathological prognostic factors (n = 178)

characteristics	Hazard ratio	95% confidence interval	p value*
*disease-free survival (DFS)*			
Age (>/< median)	0.912	0.509-1.633	n.s.
FIGO stage (III/IV vs. II vs. I)	2.112	1.486-3.002	**< 0.001**
Nodal involvement (N1 vs. N0)	3.125	1.750-5.579	**< 0.001**
Grading (G3 vs. G1/G2)	1.164	0.656-2.066	n.s.
histological type (adenomatous/adenosquamous vs. squamous)	1.118	0.597-2.093	n.s.
lymphatic invasion (L1 vs. L0)	2.934	1.300-6.621	**0.010**
depth of invasion (> 10 mm vs. 1-10 mm)	3.303	1.448-7.531	**0.004**
Adjuvant therapy (radiation vs. none)	1.834	0.961-3.499	0.066
Adjuvant therapy (chemoradiation vs.nNone)	0.743	0.325-1.699	n.s.
ALCAM IHC (positive vs. negative)	0.912	0.509-1.633	n.s.

*cancer-specific survival (CSS)*			

Age (>/< median)	1.011	0.472-1.638	n.s.
FIGO stage (III/IV vs. II vs. I)	2.265	1.555-3.299	**< 0.001**
Nodal involvement (N1 vs. N0)	3.664	1.972-6.811	**< 0.001**
Grading (G3 vs. G1/G2)	1.325	0.715-2.457	n.s.
histological type (adenomatous/adenosquamous vs. squamous)	1.012	0.507-2.020	n.s.
lymphatic invasion (L1 vs. L0)	2.960	1.232-7.113	**0.015**
depth of invasion (> 10 mm vs. 1-10 mm)	3.221	1.325-7.831	**0.010**
Adjuvant therapy (radiation vs. none)	1.840	0.930-3.643	0.080
Adjuvant therapy (chemoradiation vs. none)	0.674	0.266-1.713	n.s.
ALCAM IHC (positive vs. negative)	0.880	0.472-1.638	n.s.

*significant p-values are shown in bold

**Figure 3:**
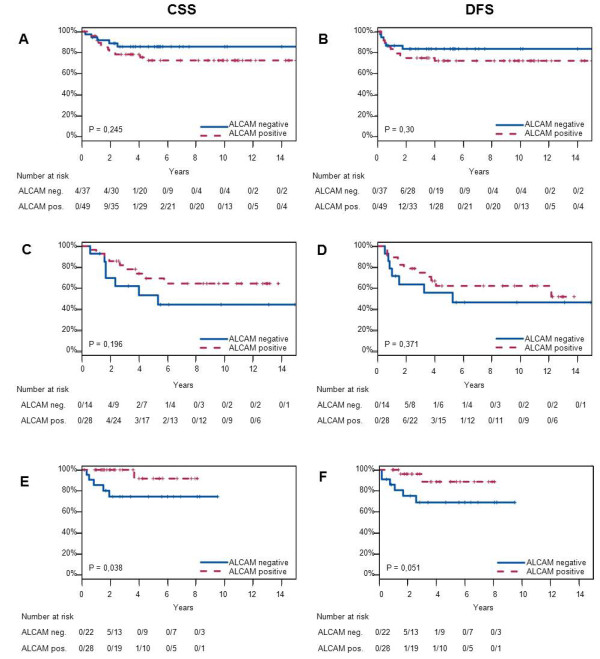
**Survival analysis of low (IRS 0-1) and high (IRS 2-12) ALCAM staining**.

After stratification according to the applied adjuvant therapy, untreated patients with ALCAM expression appeared to have a shorter DFS and CSS, yet these differences were not significant (Figure 3A, B). In patients receiving radiotherapy, a tendency into the opposite direction was observed, since ALCAM expression was associated with prolonged survival in this group (Figure 3C, D). In patients who were treated with chemoradiation (Figure 3E, F) this effect was most prominent, with a significantly longer CSS (cumulative incidence rates at 96 months 8%, 95% CI 0%-23%, and 26%, CI 3%-43%, p = 0.038) and DFS (cumulative incidence rates at 96 months 11%, CI 0%-26%, and 31%, CI 6%-49%, p = 0.051).

A and B, CSS and DFS in patients without further treatment other than tumor excision (n = 86). C and D, CSS and DFS in patients who received radio therapy after tumor excision (n = 42). E and F, CSS and DFS in patients who received chemo radiation after operation (n = 50)

In addition to ALCAM, other prognostic factors like FIGO-stage, lymph node involvement, grading, and preoperative metastasis could also influence patients' outcome. A multivariate analysis could not be performed due to the small size of the observed subgroups. We therefore analysed the distribution of these clinicopathological markers in each therapy group and in the subgroups showing low and high ALCAM expression. By this approach we could demonstrate that histopathological and clinical tumor characteristics were similarly distributed between all groups, indicating that the predictive effect of ALCAM expression was independent and not due to associations with known prognostic parameters in subgroups (Additional file 3).

### S-ALCAM levels in serum of cervical cancer patients

s-ALCAM levels were analysed by ELISA in a subset of patients (n = 55). The median s-ALCAM level was 27.6 ng/ml (range 17.5-55.1 ng/ml, mean 28.9 ng/ml). When comparing the s-ALCAM levels to ALCAM expression levels in cervical carcinoma tissue no significant associations were revealed (p = 0.515). We also correlated s-ALCAM levels to clinicopathological factors as described for ALCAM expression in tumor tissue. It could be shown that moderately differentiated tumors (G2) showed significantly higher s-ALCAM levels compared to poorly differentiated carcinomas G3 (17.4% increase, 95% CI 2.4%-29.7%, p = 0.016). G1 tumors were excluded because of the low case number (n = 2). However, no other significant associations could be found and s-ALCAM levels were not associated with patients' outcome (not shown).

## Discussion

In this hypothesis generating study ALCAM and s-ALCAM expression levels in tumor tissue and sera of cervical carcinoma patients were evaluated for the first time. Here we were able to show that ALCAM expression in tumor cells might be a predictive marker for response to chemoradiation in this cancer entity.

By evaluating ALCAM reactivity in tumor cells we were able to show that ALCAM expression is present in a substantial portion of tumors but does not correlate with prognostic markers like clinical stage, grading, age or histological type, or with patients' survival. Although few other groups distinguish between cytoplasmic and membranous ALCAM expression in neoplastic tissue following the idea that a stronger cytoplasmic expression pattern might be abnormal and associated with tumor progression [19-21], we refrained from separating ALCAM expression in membraneous and cytoplasmic staining for two reasons: first we found that cytoplasmic and membranous ALCAM staining correlated positively with each other, second, in cases of intense ALCAM expression it is often difficult to assess the staining intensity of each compartment properly (Additional file 1: Figure S1 and unpublished data).

Prior studies in other cancer entities have reported that ALCAM is upregulated in some and downregulated in others [8]. Contrary to our results high ALCAM expression in the primary tumor was found to be associated with reduced survival or unfavourable prognostic markers in some tumor types, i.e. colorectal [4,7], oral [10,21], esophageal [11], pancreatic [22] and gastric carcinomas [23] as well as neuroblastoma [24]. However, similar to previous studies in breast cancer, our results indicate that ALCAM might represent a predictive marker in cervical cancer: In patients who did not receive any further therapy after tumor excision, high ALCAM expression levels were associated with shorter CSS and DFS, whereas in patients receiving adjuvant chemoradiation high ALCAM expression was indicative of a better prognosis. In a prior study in breast cancer patients we analysed ALCAM expression in tumor tissues on protein and mRNA level using c-DNA microarray data and western blot analysis [12]. Similar to the present data obtained in cervical carcinomas, ALCAM expression was not relevant for prognosis in the total breast cancer cohort. However, in the subgroup of chemotherapy-treated patients, high ALCAM expression levels were associated with significantly longer DFS and CSS. These results could be at least partly explained by our experimental study where ALCAM expression was increased or silenced by stable transfection in breast cancer cell lines: In those cells, the consequences of high ALCAM expression were complex and included enhanced invasive potential (which might result in a more aggressive tumor growth) and increased apoptosis (which might lead to higher chemosensitivity and a better prognosis in chemotherapy-treated patients) [25]. In pancreatic cancer cells, a reduced ALCAM expression has also been shown to be associated with chemoresistance, in that case to gemcitabine and actinomycin D [13]. The results of our present study suggest that the consequences of high ALCAM expression might be similar in cervical cancer cells.

Comparison of the subcohorts treated with radiotherapy alone and with chemoradiation suggests that ALCAM expression is more predictive for successful chemotherapy compared to radiation. The mechanisms of resistance to radiotherapy and resistance to chemotherapy share similar features (role of apoptosis) as well as different characteristics (i.e. drug activation and accumulation in chemoresistance, and DNA repair mechanisms in radioresistance). Our results indicate that both mechanisms might be influenced by ALCAM in different, as yet unknown ways.

Since ALCAM is shedded into the blood by proteases, we also analysed s-ALCAM levels in a subcohort of 55 patients. The resulting s-ALCAM levels in our patients (mean 28.9 ng/ml) are below those reported for breast cancer patients (74 ng/ml; [15]) and ovarian cancer patients (mean 44 ng/ml; [14]), but slightly above those found in patients with esophageal carcinoma (mean 23.9 ng/ml; [26]). Healthy control groups showed mean s-ALCAM levels of 60 ng/ml [15], 20.6 ng/ml [26] and 29 ng/ml [14], respectively. During our experiments, there was no control group available which was comparable to the patients with respect of sex, age and sample storage. Yet, the low s-ALCAM levels in our cervical cancer cohort and the lack of association of s-ALCAM values with ALCAM immunostaining in cancer tissue or patients' outcome suggests that s-ALCAM is not an acceptable serum biomarker in this tumor type.

For squamous cell carcinomas of the cervix or other sites there are only a few studies available, which provide predictive markers for response to radiotherapy or chemotherapy, mainly by analysing cDNA expression arrays [27-29]. Adhesion molecules have been identified to be involved in chemoresistance in some prior studies on different cancer types: In neuroblastoma cell lines, acquired resistance to vincristine and doxorubicine was associated with downregulation of the neural cell adhesion molecule (NCAM) [30]. Similarly, paclitaxel-resistant ovarian carcinoma cell lines showed a decreased expression of the epithelial adhesion molecule E-cadherin [31]. ALCAM silencing has been shown to induce chemoresistance in pancreatic cancer cells in vitro [13]. In breast cancer patients [12] and in this study analysing cervical cancer patients, ALCAM expression in cancer tissue correlates with increased sensitivity to chemoradiation or chemotherapy which might at least partly result from its influence on apoptosis [25].

Limitations of this study are its retrospective design and the heterogeneous group of patients with relatively small numbers in the treatment groups. However, the heterogeneity in this first, hypothesis-generating study on ALCAM expression in cervical cancer led to interesting data which suggest that the impact of ALCAM overexpression might be treatment-dependent. To further investigate the potential predictive value of ALCAM expression for chemoradiation in cervical cancer, it will be necessary to investigate ALCAM expression levels in a prospective manner, using i.e. tissue biopsies of patients with advanced disease (FIGO III/IV) to analyse outcome after chemoradiation.

## Conclusion

In summary we hypothesize, that ALCAM expression in cervical carcinoma might be associated with improved chemoradiation response. Although the exact underlying mechanisms remain unclear, these results might help to stratify patients, who would benefit from a combined chemoradiation therapy in cervical cancer.

## Competing interests

The authors declare that they have no competing interests.

## Authors' contributions

MI and KML participated in the design of the study. KK carried out the immunohistochemistry and ELISA experiments. EK and MC participated in histological diagnosis and evaluation of IHC results. JFK performed the statistical analysis. HZ helped with establishment of the ELISA tests. VM, LW, SM, KK and FJ contributed to tissue sampling and collection of clinical data. MI, KML and LW drafted the manuscript. All authors read and approved the final manuscript.

## Pre-publication history

The pre-publication history for this paper can be accessed here:

http://www.biomedcentral.com/1471-2407/12/140/prepub

## Supplementary Material

Additional file 1 **Figure S1: Cytoplasmic and membraneous ALCAM reactivity**. Examples of ALCAM immunostaining in three cervical carcinomas showing concomitant cytoplasmic and membraneous ALCAM reactivity in tumor cells. Click here for file

Additional file 2 **Table S1: Multivariate analysis in the total cohort**. The table shows a Cox regression analysis for disease-free survival (DFS) and cancer-specific survival (CSS) in the total cohort including FIGOn stage, nodal involvement, grading, histological type, and ALCAM immunoreactivity. Click here for file

Additional file 3 **Table S2: Distribution of clinical and histological prognostic factors among subcohorts of cervical cancer patients**. After stratification according to the applied therapy, a multivariate analysis could not be performed due to the small subgroup sizes. We therefore analysed the distribution of the clinicopathological prognostic markers in each therapy group and in the subgroups showing low and high ALCAM expression. By this approach we could demonstrate that histopathological and clinical tumor characteristics were similarly distributed between all groups, indicating that the predictive effect of ALCAM expression in the chemoradiation group was independent. Click here for file
