# Supplementary material for: Relevance of activated leukocyte cell adhesion molecule (ALCAM) in tumor tissue and sera of cervical cancer patients
Source: BMC Cancer. 2012 Apr 4;12:140. doi: 10.1186/1471-2407-12-140 (PMC3348036; doi:10.1186/1471-2407-12-140)
Supplement: Additional file 1 — Figure S1: Cytoplasmic and membraneous ALCAM reactivity. Examples of ALCAM immunostaining in three cervical carcinomas showing concomitant cytoplasmic and membraneous ALCAM reactivity in tumor cells. [file 1471-2407-12-140-S1.PPT]

## Slide 1
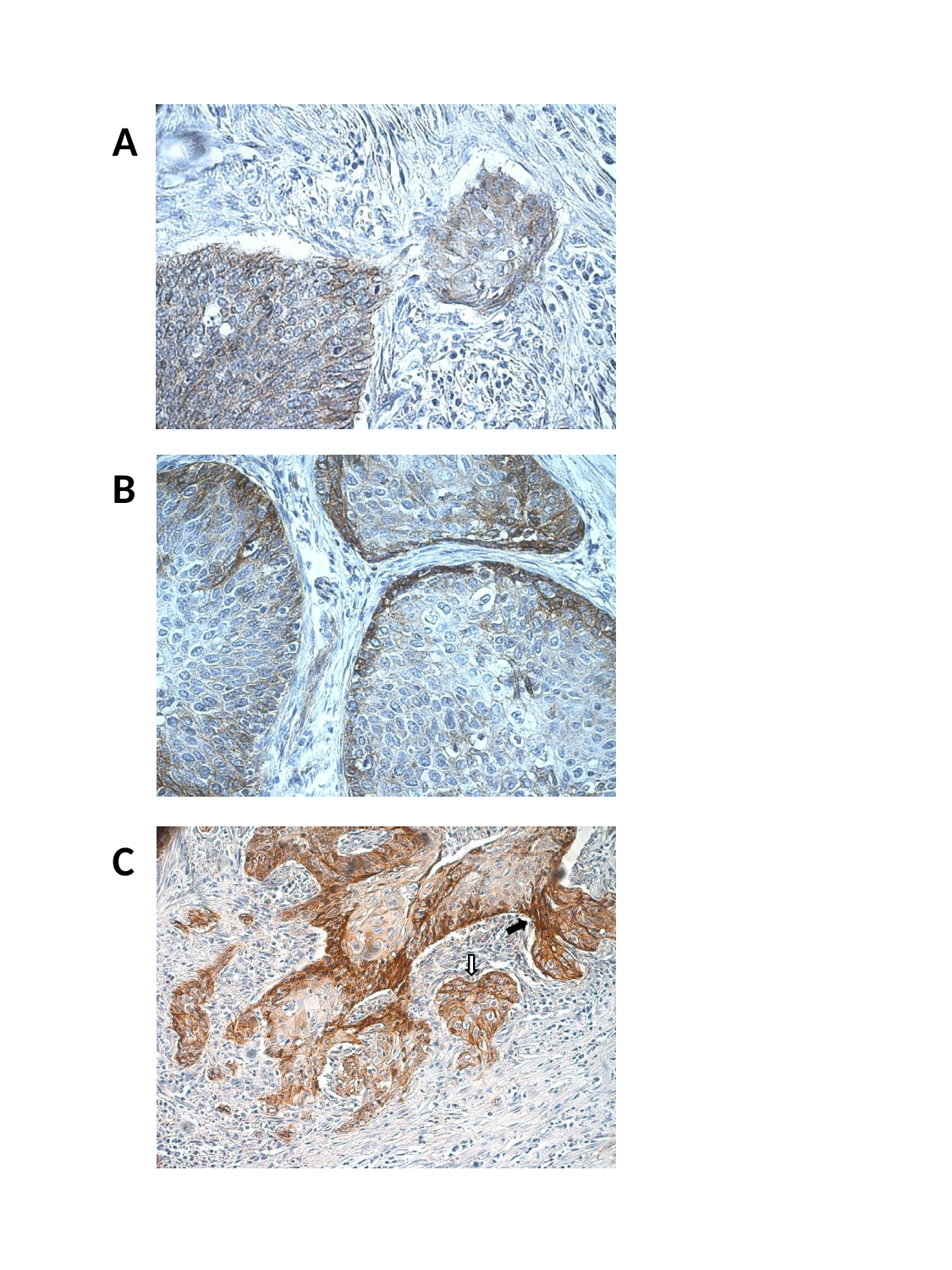

A
B
C

## Slide 2
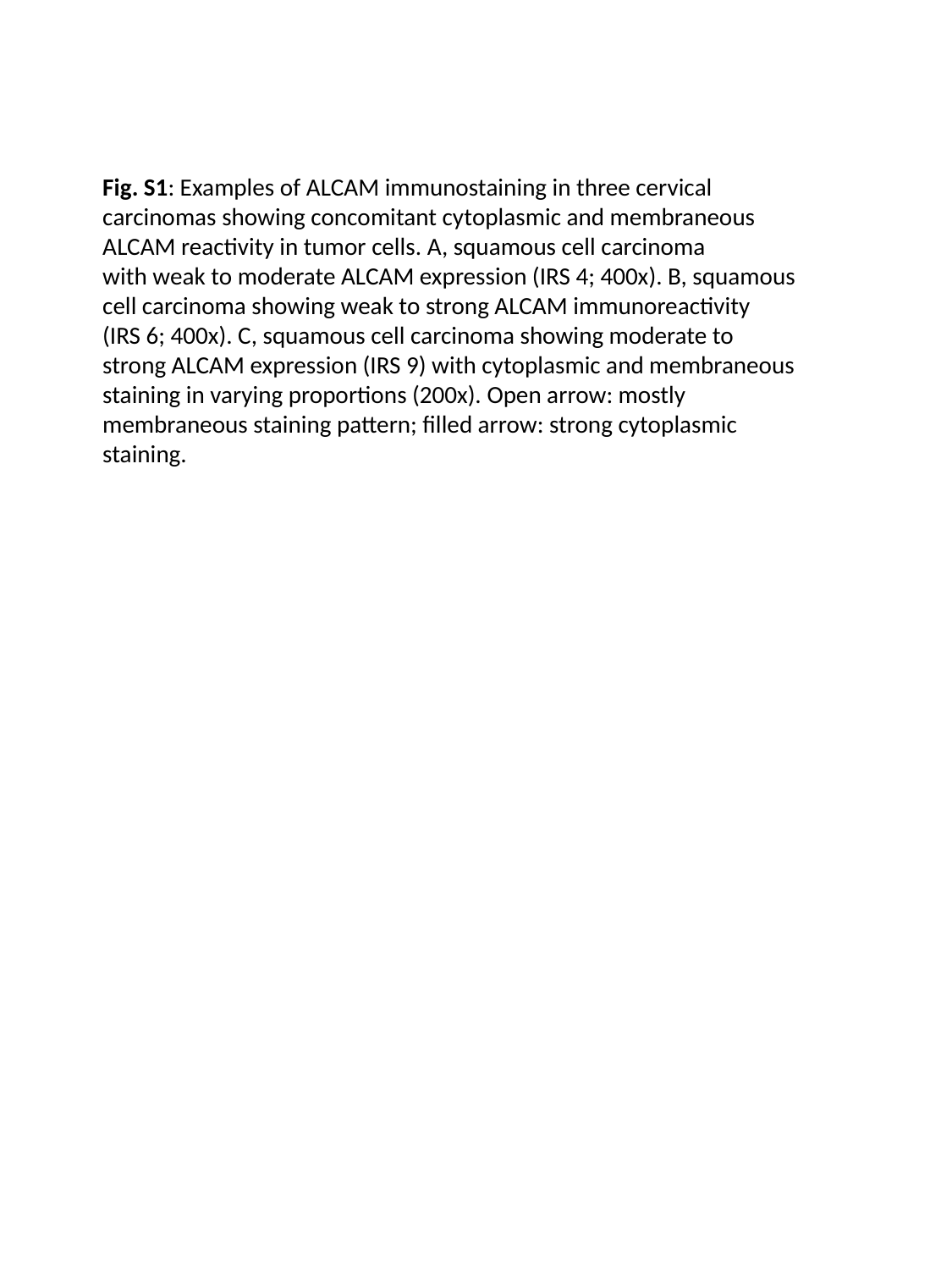

Fig. S1: Examples of ALCAM immunostaining in three cervical
carcinomas showing concomitant cytoplasmic and membraneous
ALCAM reactivity in tumor cells. A, squamous cell carcinoma
with weak to moderate ALCAM expression (IRS 4; 400x). B, squamous
cell carcinoma showing weak to strong ALCAM immunoreactivity
(IRS 6; 400x). C, squamous cell carcinoma showing moderate to
strong ALCAM expression (IRS 9) with cytoplasmic and membraneous
staining in varying proportions (200x). Open arrow: mostly
membraneous staining pattern; filled arrow: strong cytoplasmic
staining.
