# Supplementary material for: Relevance of activated leukocyte cell adhesion molecule (ALCAM) in tumor tissue and sera of cervical cancer patients
Source: BMC Cancer. 2012 Apr 4;12:140. doi: 10.1186/1471-2407-12-140 (PMC3348036; doi:10.1186/1471-2407-12-140)
Supplement: Additional file 2 — Table S1: Multivariate analysis in the total cohort. The table shows a Cox regression analysis for disease-free survival (DFS) and cancer-specific survival (CSS) in the total cohort including FIGOn stage, nodal involvement, grading, histological type, and ALCAM immunoreactivity. [file 1471-2407-12-140-S2.DOCX]

| **Table S1: Multivariate analysis in the total cohort** |  |  |  |
| --- | --- | --- | --- |
| characteristics | Hazard ratio | 95% confidence interval | p value* |
| *disease-free survival (DFS)* |  |  |  |
|  |  |  |  |
| FIGO stage (III/IV vs. II vs. I) | 1.744 | 1.182 – 2.574 | **0.005** |
| Nodal involvement (N1 vs. N0) | 2.125 | 1.107 – 4.080 | **0.024** |
| Grading (G3 vs. G1/G2) | 1.208 | 0.714 - 2.044 | 0.482 |
| histological type (adenomatous/adenosquamous vs. squamous) | 0.962 | 0.501 – 1.844 | 0.906 |
| ALCAM IHC (positive vs. negative) | 0.841 | 0.463 – 1.528 | 0.570 |
|  |  |  |  |
| *cancer-specific survival (CSS)* |  |  |  |
|  |  |  |  |
| FIGO stage (III/IV vs. II vs. I) | 1.832 | 1.216 – 2.762 | **0.004** |
| Nodal involvement (N1 vs. N0) | 2.592 | 1.304 – 5.151 | **0.007** |
| Grading (G3 vs. G1/G2) | 1.479 | 0.824 - 2.654 | 0.190 |
| histological type (adenomatous/adenosquamous vs. squamous) | 0.835 | 0.406 – 1.716 | 0.624 |
| ALCAM IHC (positive vs. negative) | 0.808 | 0.425 - 1.534 | 0.514 |
|  |  |  |  |
| *significant p-values are shown in bold. |  |  |  |
|  |  |  |  |
