# Supplementary material for: Relevance of activated leukocyte cell adhesion molecule (ALCAM) in tumor tissue and sera of cervical cancer patients
Source: BMC Cancer. 2012 Apr 4;12:140. doi: 10.1186/1471-2407-12-140 (PMC3348036; doi:10.1186/1471-2407-12-140)
Supplement: Additional file 3 — Table S2: Distribution of clinical and histological prognostic factors among subcohorts of cervical cancer patients. After stratification according to the applied therapy, a multivariate analysis could not be performed due to the small subgroup sizes. We therefore analysed the distribution of the clinicopathological prognostic markers in each therapy group and in the subgroups showing low and high ALCAM expression. By this approach we could demonstrate that histopathological and clinical tumor characteristics were similarly distributed between all groups, indicating that the predictive effect of ALCAM expression in the chemoradiation group was independent. [file 1471-2407-12-140-S3.DOCX]

| **Table S2: Distribution of clinical and histological prognostic factors among subcohorts of cervical cancer patients** | | | | | | | |
| --- | --- | --- | --- | --- | --- | --- | --- |
|  |  |  |  |  |  |  |  |
|  | **ALCAM expression** | | | | |  |  |
|  | negative (IRS 0–1) | |  | positive (IRS 2–12) | |  |  |
|  | n | (%) |  | n | (%) |  | p value |
| **Patients without adjuvant therapy** |  |  |  |  |  |  |  |
| All patients in subcohort | 37 | 100 |  | 49 | 100 |  |  |
|  |  |  |  |  |  |  |  |
|  |  |  |  |  |  |  |  |
| FIGO stage |  |  |  |  |  |  | 0.838^a^ |
| I | 28 | 76 |  | 38 | 78 |  |  |
| II | 6 | 16 |  | 7 | 14 |  |  |
| III/IV | 3 | 8 |  | 4 | 8 |  |  |
|  |  |  |  |  |  |  |  |
| Nodal involvement |  |  |  |  |  |  | 0.649 |
| pN0 | 35 | 95 |  | 44 | 90 |  |  |
| pN1 | 2 | 5 |  | 5 | 10 |  |  |
|  |  |  |  |  |  |  |  |
| Preperative metastasis |  |  |  |  |  |  | 0.456 |
| pM0 | 36 | 97 |  | 46 | 94 |  |  |
| pM1 | 1 | 3 |  | 3 | 6 |  |  |
|  |  |  |  |  |  |  |  |
| Grading |  |  |  |  |  |  | 0.436^b^ |
| G1 | 2 | 5 |  | 3 | 6 |  |  |
| G2 | 20 | 54 |  | 22 | 45 |  |  |
| G3 | 15 | 41 |  | 24 | 49 |  |  |
|  |  |  |  |  |  |  |  |
| **Patients with adjuvant radiotherapy** |  |  |  |  |  |  |  |
| All patients in subcohort | 14 | 100 |  | 28 | 100 |  |  |
|  |  |  |  |  |  |  |  |
| FIGO stage |  |  |  |  |  |  | 1.000^a^ |
| I | 5 | 36 |  | 10 | 36 |  |  |
| II | 5 | 36 |  | 11 | 39 |  |  |
| III/IV | 4 | 28 |  | 7 | 25 |  |  |
|  |  |  |  |  |  |  |  |
| Nodal involvement |  |  |  |  |  |  | 0.382 |
| pN0 | 8 | 57 |  | 12 | 43 |  |  |
| pN1 | 6 | 43 |  | 16 | 57 |  |  |
|  |  |  |  |  |  |  |  |
| Preperative metastasis |  |  |  |  |  |  | 0.558 |
| pM0 | 11 | 79 |  | 24 | 86 |  |  |
| pM1 | 3 | 21 |  | 4 | 14 |  |  |
|  |  |  |  |  |  |  |  |
| Grading |  |  |  |  |  |  | 0.381^b^ |
| G1 | 0 | 0 |  | 1 | 4 |  |  |
| G2 | 9 | 64 |  | 13 | 46 |  |  |
| G3 | 5 | 36 |  | 14 | 50 |  |  |
|  |  |  |  |  |  |  |  |
| **Patients with adjuvant chemoradiation** |  |  |  |  |  |  |  |
| All patients in subcohort | 22 | 100 |  | 28 | 100 |  |  |
|  |  |  |  |  |  |  |  |
| FIGO stage |  |  |  |  |  |  | 0.374^a^ |
| I | 9 | 41 |  | 15 | 54 |  |  |
| II | 9 | 41 |  | 11 | 39 |  |  |
| III/IV | 4 | 18 |  | 2 | 7 |  |  |
|  |  |  |  |  |  |  |  |
| Nodal involvement |  |  |  |  |  |  | 0.336 |
| pN0 | 12 | 55 |  | 19 | 68 |  |  |
| pN1 | 10 | 45 |  | 9 | 32 |  |  |
|  |  |  |  |  |  |  |  |
| Preperative metastasis |  |  |  |  |  |  | 0.558 |
| pM0 | 19 | 86 |  | 26 | 93 |  |  |
| pM1 | 3 | 14 |  | 2 | 7 |  |  |
|  |  |  |  |  |  |  |  |
| Grading |  |  |  |  |  |  | 0.585^b^ |
| G1 | 1 | 5 |  | 0 | 0 |  |  |
| G2 | 6 | 27 |  | 11 | 39 |  |  |
| G3 | 15 | 68 |  | 17 | 61 |  |  |
